# Psychological stress of emergency medical staff after the largest mass murder incident in post‐World War II era

**DOI:** 10.1002/npr2.12237

**Published:** 2022-02-16

**Authors:** Hisatoshi Arai, Katsuo Inoue, Takeya Takizawa, Tatsuhiro Yamaya, Yasushi Asari, Hitoshi Miyaoka

**Affiliations:** ^1^ 13031 Department of Psychiatry Saitama Medical University Iruma‐gun Japan; ^2^ 38088 Department of Psychiatry Kitasato University School of Medicine Sagamihara Japan; ^3^ 38088 Department of Critical Care and Emergency Medicine Kitasato University School of Medicine Sagamihara Japan

**Keywords:** critical incident stress, emergency and disaster medical center, IES‐R, mental health of medical staff, posttraumatic stress disorder (PTSD)

## Abstract

**Background:**

A mass‐casualty incident occurred on July 26, 2016, at Tsukui Yamayuri‐en, which is a welfare facility for people with intellectual disabilities. Nineteen residents with intellectual disabilities were killed, and 26 other residents and staff members were injured. Kitasato University Hospital Emergency and Disaster Medical Center treated many patients in serious condition at the site and in the hospital.

**Aims:**

The authors investigated the symptom severity and distributions of posttraumatic stress disorder (PTSD) among the emergency medical staff in charge.

The subjects of this study were the staff members, mostly working at the Emergency and Disaster Medical Center, who treated the people injured in the incident.

**Methods:**

We conducted a questionnaire survey using Impact of Event Scale‐Revised (IES‐R) on 104 staff members, and 79 responded.

**Results:**

The IES‐R scores of nurses were significantly higher than those of doctors. There was no significant difference in the scores between male and female staff members, and there was no correlation between the score of each IES‐R subscale and age.

**Conclusion:**

Results suggest that mental care should be provided to emergency medical staff, especially nurses who treat trauma patients involved in disasters and heinous crimes.

## Abstract 2

Mental care should be provided to emergency medical staff, especially nurses who treat trauma patients involved in disasters and heinous crimes.

## Graphical abstract

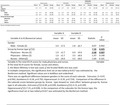

## 1 INTRODUCTION

A mass‐casualty incident occurred on July 26, 2016, at Tsukui Yamayuri‐en, which is a welfare facility for people with intellectual disabilities. Nineteen residents with intellectual disabilities were killed, and 26 other residents and staff members were injured. The number of people who were killed was the largest in post‐World War II Japan. Kitasato University Hospital Emergency and Disaster Medical Center dispatched their doctor cars to the site of the incident and treated many patients with severe injury and in serious condition at the site and in the hospital. The authors investigated the symptom severity and distributions of posttraumatic stress disorder (PTSD) among the emergency medical staff in charge.

Critical incident stress (CIS) is defined as “a stress reaction of a person who has faced an incident or threat where the normal coping mechanism cannot work.”^1^ Rescuers and helpers who respond to sudden mass murder incidents and large‐scale disasters may experience CIS.

It is important to consider the CIS of emergency and disaster relief workers who rescue or provide medical care to victims of large‐scale disasters or mass‐casualty crimes and who hold major responsibilities. They often suffer from posttraumatic stress disorder (PTSD), which continues for a long duration.

There are few reports on the stress experienced by their mental health of medical and emergency medical staff providing medical care at the scenes of large‐scale disasters or mass‐casualty incidents. Investigating the level of psychological stress of emergency medical staff, however, is important for them to be able to maintain their good mental health. We investigated the stress and mental health of the medical and emergency medical staff involved in treating the victims of the above incident.

## 2 METHODS

### 2.1 Subjects

The subjects of this study were the staff of Kitasato University Hospital working at the Emergency and Disaster Medical Center, who treated the people injured in the murder incident that occurred at Tsukui Yamayuri‐en care facility on July 26, 2016.

The center is located in Sagamihara City in the northern part of Kanagawa Prefecture, Japan, and is the only third‐level emergency hospital in the city. As an emergency hospital, the center covers a total population of more than 1.5 million people. We conducted a questionnaire survey of 104 staff members, and 79(76.0%)responded from August 1, to August 31, 2016. This study was approved by the Ethics Committee of Kitasato University School of Medicine on September 1, 2017, Number: B 16‐229, and written informed consent was obtained from all the subjects. This study has been performed according to the Declaration of Helsinki.

### 2.2 Measures

The Japanese translated version of Impact of Event Scale‐Revised (IES‐R‐J) was used in the survey. IES‐R is a self‐report questionnaire developed by Weiss and Marmar^2^ for the purpose of assessing PTSD symptoms. IES‐R consists of 22 items divided into three subscales, namely intrusive recollections (hereafter, intrusion), avoidance, and hyperarousal, which are three major PTSD symptoms^2^, ^3^The subjects were asked to rate the severity of symptoms over the past week on a five‐point scale (0‐4) for each of the 22 items. Those with an IES‐R total score of 25 or higher (0‐88 points) are considered likely to develop PTSD^3^, ^4^

### 2.3 Statistical analysis

Background characteristics such as gender and license type (for classifying the subjects as physicians, nurses and others) of the subject were summarized. Furthermore, age and the IES‐R scores for each subscale were analyzed. Data were analyzed by tests for unmatched pairs using statistical significance tests. To compare the difference for unmatched pairs, the score of each IES‐R subscale was compared between genders using the Mann‐Whitney U test. The Kruskal‐Wallis test was used in the comparison of the score of each IES‐R subscale between license types and the Mann‐Whitney U test for multiple comparisons. The relationship between the age and the score of each IES‐R subscale was analyzed using Spearman's rank correlation coefficient.

For the interpretation of results, the significance probability for each analysis was corrected by the Bonferroni method as a multiple adjustment method. The statistical analyses were performed using SPSS 22.0 (IBM SPSS Inc2013).

## 3 RESULTS

### 3.1 Background characteristics

The background characteristics of the subjects are shown in Table 1. The numbers of male and female subjects were 50 (60.3%) and 29 (36.7%), respectively.

**TABLE 1:** Background characteristics of subjects

				Age	
Characteristics		n	%	Mean ± SD	[Range]
All subjects		79	100	36.2 ± 9.2	[22‐62]
Gender	Male	50	63.3	37.2 ± 7.9	[25‐56]
	Female	29	36.7	34.2 ± 11.0	[22‐62]
Group by license type	Physician (M = 19,F = 6)	25	31.6	36.9 ± 8.0	[25‐55]
	Nurse (M = 7,F = 9)	16	20.3	36.9 ± 9.2	[27‐62]
	Others (M = 24,F = 14)	38	48.1	35.4 ± 10.0	[22‐56]

Note

Nurses included assistant nurses.

The numbers of subjects grouped by their license type were 25 (31.6%) for physicians, 16 (20.2%) for nurses, including assistant nurses, and 38 (48.1%) for others. The average age of the physicians, nurses, and others was approximately 36 years.

### 3.2 IES‐R scores by age and license type

IES‐R total scores by age are shown in Table 2. The average total scores of the subjects were 1.69 ± 2.97 for those aged 20 to 29, 3.17 ± 5.93 for those aged 30 to 39, 3.55 ± 9.22 for those aged 40 to 49, and 9.00 ± 13.14 for those aged 50 and over.

**TABLE 2:** IES‐R total score by age

Age(years)	Median	Range	Mean ± SD
20‐29 years (n = 26)	1	[0‐13]	1.69 ± 2.97
30‐39 years (n = 24)	0	[0‐23]	3.17 ± 5.93
40‐49 years (n = 20)	0	[0‐40]	3.55 ± 9.22
50 or older (n = 7)	3	[0‐39]	9.0 ± 13.14

The total score of all IES‐R subscales and the score of each IES‐R subscale for genders and license types are shown in Table 3. The mean IES‐R total scores for males and females were 3.4 ± 7.5 and 2.8 ± 6.7, respectively. The mean IES‐R total scores for the subjects grouped by license type were 1.0 ± 2.7 for physicians, 6.0 ± 9.0 for nurses, and 3.5 ± 8.0 for others. Among them, one nurse and one person in the group with other license types were identified as high‐risk individuals on the basis of their IES‐R scores. In the grouping according to license type, the mean subscores for intrusion, which is a subscale of IES‐R, were 0.3 ± 0.9 for physicians, 2.8 ± 4.3 for nurses, and 1.2 ± 2.5 for others. The avoidance subscores were 0.4 ± 1.3 for physicians, 2.1 ± 3.8 for nurses, and 1.4 ± 3.9 for others. The hyperarousal subscores were 0.2 ± 0.8 for physicians, 1.1 ± 1.5 for nurses, and 1.0 ± 2.5 for others.

**TABLE 3:** Scores of IES‐R subscales for characteristics

**Subscales**												
Characteristics	Total scores			Intrusion			Avoidance			Hyperarousal		
	Mean ± SD	Median	Range	Mean ± SD	Median	Range	Mean ± SD	Median	Range	Mean ± SD	Median	Range
**All subjects**	3.2 ± 7.2	0.0	[0‐40]	1.2 ± 2.7	0.0	[0‐16]	1.2 ± 3.3	0.0	[0‐18]	0.8 ± 1.9	0.0	[0‐11]
**Gender**												
**Male**	3.4 ± 7.5	0.0	[0‐40]	1.2 ± 2.5	0.0	[0‐12]	1.4 ± 3.6	0.0	[0‐18]	0.9 ± 2.3	0.0	[0‐11]
**Female**	2.8 ± 6.7	0.0	[0‐34]	1.3 ± 3.2	0.0	[0‐16]	0.9 ± 2.7	0.0	[0‐14]	0.6 ± 1.3	0.0	[0‐4]
**Group by license type**												
**physician**	1.0 ± 2.7	0.0	[0‐13]	0.3 ± 0.9	0.0	[0‐4]	0.4 ± 1.3	0.0	[0‐5]	0.2 ± 0.8	0.0	[0‐4]
**nurse**	6.0 ± 9.0	2.0	[0‐34]	2.8 ± 4.3	0.5	[0‐16]	2.1 ± 3.8	0.0	[0‐14]	1.1 ± 1.5	0.5	[0‐4]
**Others**	3.5 ± 8.0	0.0	[0‐40]	1.2 ± 2.5	0.0	[0‐12]	1.4 ± 3.9	0.0	[0‐18]	1.0 ± 2.5	0.0	[0‐11]

Note

Nurses included assistant nurses.

### 3.3 Comparison of IES‐R score between genders and groups by license type

The IES‐R total scores for genders and license types are shown in Table 4. There was no significant difference in the IES‐R total score between males and females (Z = −0.07, *P* = .943). The main effect was recognized in the IES‐R total score for license type (χ^2^ = 7.35, *P* = .025), and multiple comparisons showed that the IES‐R total score of nurses was significantly higher than that of physicians (Z = −2.68, *P* = .007). In all subscales, there was no significant difference between genders, and no main effect between groups by license type was observed. On the contrary, there was no correlation between the score of each IES‐R subscale and age (Table 5).

**TABLE 4:** Total IES‐R scores for genders and license types

	Variable A	Variable B		
Variable A to B (Numerical value)	Mean ± SD	Mean ± SD	Statistic	*P*‐value
Gender				
Male ‐ Female (Z)	3.4 ± 7.5	2.8 ± 6.7	−0.07	0.943
Group by license type (χ^2^[2])			**7.35**	**0.025**
Physicians ‐ Nurses (Z)	1.0 ± 2.7	6.0 ± 9.0	−**2.68**	**0.007**
Physicians ‐ Others (Z)	1.0 ± 2.7	3.5 ± 8.0	−1.47	0.140
Nurses ‐ Others(Z)	6.0 ± 9.0	3.5 ± 8.0	−1.64	0.101

Note

Variable A: The total IES‐R scores for male, physicians, and nurses.

Variable B: The total IES‐R scores for female, nurses, and others.

Z, the Mann‐Whitney U test was used; χ^2^, the Kruskal‐Wallis test was used.

In multiple comparisons, the significance level set at two‐tailed *P* < .017 was estimated by the Bonferroni method. Significant values are in boldface and underlined.

There was no significant difference between genders in the score of each subscale (Intrusion: Z=−0.07, *P* =.942; Avoidance: Z = −0.26, *P* = .792; Hyperarousal: Z = −0.35, *P* = .724). Comparison of the difference in the subscale scores between groups by license type showed no main effect between groups by license type in all subscales. (Intrusion: χ^2^[2] = 6.49, *P* = .039; Avoidance: χ^2^[2] = 3.13, *P* = .209; Hyperarousal: χ^2^[2] = 7.15, *P* = .028). In the comparison of the subscales for the license type, the significance level set at two‐tailed *P* < .017 was estimated by the Bonferroni method.

**TABLE 5:** Relationship between scores of subscales and age

	r_s_	*P*‐value
Total score of IES‐R	0.04	0.759
Scores of subscales		
Intrusion	0.12	0.311
Avoidance	0.11	0.335
Hyperarousal	0.12	0.311

Abbreviations: IES‐R, Impact of Event Scale‐Revised; r_s_, Spearman's rank correlation coefficient.

## 4 DISCUSSION

### 4.1 Psychological stress of emergency medical staff

There was no significant gender difference in the IES‐R total score. In general, the risk of developing PTSD is higher in females than in males^5^, ^6^ however, a report showed that there is no significant gender difference in the risk of developing PTSD after natural disasters and accidents and that the risk tends to be low in both males and females^7^

Age is considered to be a factor affecting coping behavior. It has been suggested that the coping mechanism becomes rigid and loses its flexibility in dealing with stress with advancing age^8^ In this study, however, there was no correlation between each IES‐R score and age. Although young medical staff account for a high proportion of emergency medical staff, it is also important to provide mental care to older staff.

The IES‐R scores of nurses were significantly higher than those of doctors. Although there are few reports on the mental health of doctors in Japan, there is one report about the traumatic stress of doctors involved in prehospital emergency medical care. In that report, 46.3% of doctors had experienced being traumatized by an incident and 4.6% had an IES‐R total score of 25 or higher^9^

There have been several studies on the IES‐R scores of emergency medical staff who are involved in disaster relief in Japan. In a study on firefighters across Japan, Hatanaka et al reported that 58.1% of firefighters had experienced a shocking disaster within the past ten years and 15.6% of them showed a high risk of PTSD on the basis of their IES‐R scale scores^10^ Maruyama et al conducted a survey by sending the IES‐R questionnaire by mail to firefighters across Japan and found that 15.6% of them suffered from PTSD.^11^ It has been pointed out that emergency nurses are highly stressed in the face of job difficulties, life‐threatening job assignments, and death of patients.^12^

There have been some studies on the mental health of emergency staff in Japan focusing on firefighters and nurses; however, to the best of our knowledge, there have been few studies focusing on doctors. The results of this study may have been strongly affected by the fact that doctors working at our center have a high stress tolerance because they have encountered on a daily basis patients with serious physical injuries or patients who have attempted suicide.

The scores of the three IES‐R subscales, intrusion, avoidance, and hyperarousal, were examined in this study. Intrusion is the repeated and involuntary recall of distressing memories of a traumatic incident. Avoidance is the continual avoidance of activities, places, and physical reminders that provoke memories associated with a traumatic incident. Hyperarousal is characterized by marked changes in arousal and responsiveness associated with a traumatic incident, expressed as anger or hypervigilance. In this study, there was no significant difference in the score of each subscale between the groups according to the license type. These results may have been affected by the fact that nurses spend the longest time with patients, even in daily clinical practice, which can have a large psychological and physical burden and result in a high degree of emotional involvement. The average IES‐R scores are quite low compared with those in previous research conducted in Japan. However, some people require more mental care than others, and it may be necessary to continue supporting the mental care of the emergency medical staff for a long period of time.

### 4.2 Mental care for staff involved in emergency medical care

Emergency medical staff provide care to many people who are sent to hospital with life‐threatening traumas or other life‐threatening emergencies. Even if they can save a patient's life, they may suffer from functional impairment over a long period of time.^13^ A serious examination of the issue of mental care for emergency medical staff has only recently begun in Japan in some organizations. There is no satisfactory structure to provide them with care to cope with stress. In mass‐casualty incidents such as the Tsukui Yamayuri‐en incident, emergency medical staff see or hear about a tragic situation and are required to promptly make important decisions during triage. Because of the specificity of their jobs, emergency medical staff are subjected to particular stress at the scenes of disasters. It has been found that they are likely to exhibit posttraumatic stress reactions, including symptoms that are not serious enough to become disorders.^14^

The emergency doctors at our hospital are generally mentally resilient because they provide care to patients who have attempted suicide or patients with serious traffic injuries in their daily clinical practice. In the clinical practice of emergency medical care, they work as a team in accordance with their areas of expertise rather than alone. They can easily confer with each other. These conditions as well as their deep sense of responsibility, appropriate allocation of roles, and high level of motivation may have affected the results of this study. It has been reported that talking to others about a traumatic experience helps prevent the development of general malaise.^15^ Therefore, the development of a working environment that allows staff to express their feelings and confer with each other on a regular basis will lead to the reduction in CIS. In addition, daily conferences held in an emergency ward are very important for medical staff to share information and responsibilities with each other and to quickly respond to issues. This enables them to quickly respond to a critical incident, such as the Tsukui Yamayuri‐en incident, and to continuously provide intensive care while maintaining their high level of motivation. Moreover, most of the emergency medical staff in this study are relatively young, which may be a reason why they are adaptable to situations where speed is required. The results of this study may also reflect the fact that emergency medical staff are trained to “snap out of it” because they see patients arriving at the hospital under emergency conditions on a daily basis and they are required to treat such patients one after another.

## 5 LIMITATIONS AND FUTURE CHALLENGES

Care must be taken in interpreting the results of this study because it was conducted in a short period of time immediately after the Tsukui Yamayuri‐en incident. Also, it should be taken into account that most of the emergency medical staff in this study provided medical care not at the site of the incident but within the facilities of the hospital. It is necessary to examine in the future the late‐onset emotional reactions to a shocking incident.

## 6 CONCLUSIONS

The psychological stress experienced by the emergency medical staff who treated victims of the worst mass murder incident in post‐World War II Japan was examined. The results suggest that providing mental care is important for the emergency medical staff, especially nurses, who treat patients in a serious condition and patients with trauma caused by disasters and crimes. The results show that care should be taken to support their mental health, especially in serious events.

## CONFLICT OF INTEREST

The authors declare no conflict of interest.

## AUTHOR CONTRIBUTIONS

All authors contributed to data collection and analysis and revision of the manuscript. All authors have read and approved the final version.

## APPROVAL OF THE RESEARCH PROTOCOL BY AN INSTITUTIONAL REVIEWER BOARD

This study was approved by the Ethics Committee of Kitasato University School of Medicine on September 1, 2017, Number B 16‐229. This study was performed in accordance with the Declaration of Helsinki.

## INFORMED CONSENT

All the subjects provided written informed consent after receiving a thorough explanation of the purpose and method of this study.

## REGISTRY AND THE REGISTRATION NO. OF THE STUDY/TRIAL

Not applicable.

## ANIMAL STUDIES

Not applicable.

## Data Availability

The data are not publicly available due to privacy and ethical restrictions. The raw data belonged to the present study cannot be made publicly available, because the disclosure of personal data was not included in the research protocol of the present study.
